# Synthetic Geopolymers for Controlled Delivery of Oxycodone: Adjustable and Nanostructured Porosity Enables Tunable and Sustained Drug Release

**DOI:** 10.1371/journal.pone.0017759

**Published:** 2011-03-15

**Authors:** Johan Forsgren, Christian Pedersen, Maria Strømme, Håkan Engqvist

**Affiliations:** Department of Engineering Sciences, Uppsala University, Uppsala, Sweden; George Mason University, United States of America

## Abstract

In this article we for the first time present a fully synthetic mesoporous geopolymer drug carrier for controlled release of opioids. Nanoparticulate precursor powders with different Al/Si-ratios were synthesized by a sol-gel route and used in the preparation of different geopolymers, which could be structurally tailored by adjusting the Al/Si-ratio and the curing temperatures. In particular, it was shown that the pore sizes of the geopolymers decreased with increasing Al/Si ratio and that completely mesoporous geopolymers could be produced from precursor particles with the Al/Si ratio 2∶1. The mesoporosity was shown to be associated with a sustained and linear in vitro release profile of the opioid oxycodone. A clinically relevant release period of about 12 h was obtained by adjusting the size of the pellets. The easily fabricated and tunable geopolymers presented in this study constitute a novel approach in the development of controlled release formulations, not only for opioids, but whenever the clinical indication is best treated with a constant supply of drugs and when the mechanical stability of the delivery vehicle is crucial.

## Introduction

Chronic pain is one of the most significant health issues in the world. In the US, for example, about 30% of the population suffer from chronic pain associated with diseases such as arthritis and cancer [1]. Unsatisfactory pain management leads to a reduced quality of life for afflicted persons, and to large societal costs. Moderate to severe chronic pain is most effectively treated with opiates or with opioids, i.e. the synthetic relatives of opiates. Different strategies are employed to obtain a sustained effect from these analgesics, for example sustained release oral dosage forms [2], [3], sustained release transdermal patches [4] and implanted infusion pumps [5]. WHO recommends oral administration of analgesics in their *cancer pain guidelines* [6], since it is a non-invasive strategy which facilitates the administration and increases patient compliance. However, due to the narrow therapeutic window of opioids, “dose dumping” associated with oral administration can have fatal consequences [7]. Dose dumping is a rapid and unintended release of the entire dose from a sustained release drug carrier [8], and may occur if the carrier breaks due to mechanical stress or dissolves due to chemical reactions. It was recently suggested that geopolymers, i.e. inorganic aluminosilicate polymers, could be used in oral dosage forms to achieve sustained release in combination with mechanical strength and chemical stability of the drug carrier [9]. Geopolymers are a type of cementitious material which typically is produced by reacting fly ash or clay-derived precursor powders (e.g. metakaolin) with an alkaline sodium silicate solution [10]. The chemical stability of geopolymers is often superior to other cementitious materials such as ordinary Portland cement (OPC) [11]. In the recent study on geopolymers as drug delivery vehicles, it was shown that the delivery system offered high mechanical strength together with adjustable porosity, which enabled tuning of the drug release rate [9]. By mixing the drug, e.g. an opioid, with the precursor powder and an alkaline silicate solution during synthesis, the drug becomes integrated in the porous and mechanically strong matrix [9]. The synthesis can be performed at room temperature, which is favorable from a drug stability point of view, although it is required that the drug compound is not sensitive to the alkaline synthesis condition. The characteristics of the final product (porosity, hardness etc.) will depend on the origin of the precursor powder; different precursor powders have different Al/Si ratios and impurity contents, and differ in size and morphology. However, the surface properties, the size and the morphology of precursor particles can be controlled by synthetic precursor fabrication [12], and therefore it should be possible to optimize precursor powders for the production of sustained release geopolymers. It has hithereto not been investigated how the Al/Si ratio of the precursor powder affects the pore size distribution of the geopolymers, and how that in turn is linked to the drug release profile of the geopolymers. In the context of sustained release, it is particularly interesting to produce drug delivery systems with linear drug release profiles, i.e. zero order release, since that is expected to minimize drug concentration fluctuations in blood plasma [2]. The scope of the present study was therefore to (i) produce precursor powders of different Al/Si ratio via a sol-gel process, and produce opioid-carrying geopolymers from the precursor powders, (ii) characterize the precursor powders and the geopolymers thoroughly, and (iii) investigate if a relationship could be found between Al/Si ratio in the precursor powders and the drug release profiles of the corresponding geoplymers.

## Methods

### Precursor powders

Three different aluminosilicate powders with varying Al/Si molar ratio were prepared by precipitation from aluminum nitrate nonahydrate (ANN, Al(NO_3_)_3_· 9H_2_O, Sigma-Aldrich) and tetraethylorthosilicate (TEOS, Si(OC_2_H_5_)_4,_ Sigma-Aldrich). The sample name of each precursor powder refers to its Al/Si molar ratio, as shown in Table 1 (e.g., AS21 has an Al/Si molar ratio of 2∶1). The total concentration of ANN and TEOS together was 1.2 M in all three preparations as described elsewhere [13] (in the referred study, the synthesized aluminosilicate powders were not used as precursors in geopolymer production). For the synthesis of each powder, two solutions were prepared, *A* and *B*. Solution *A* contained TEOS diluted to 250 ml with ethanol and solution *B* contained ANN dissolved in H_2_O and diluted to 250 ml with ethanol. The TEOS/H_2_O molar ratio was 1∶18 in all preparations. The two solutions were stirred for 15 min before they were mixed together and stirred for additionally 3 h. Subsequently, 250 ml 25% ammonium hydroxide (Sigma Aldrich) was added rapidly to the mixture under vigorous stirring to cause precipitation. The obtained gels were dried on filter paper and left in a fume hood until the ammonia had evaporated, and thereafter dried at 110°C for 10 h. The dried powders were analyzed with X-ray diffraction (XRD, D5000 diffractometer - Siemens/Bruker) before subjected to a calcination at 800°C for 2 h. The powders were then again examined with XRD and the true densities (*ρ*) of the powders were examined with He-pycnometry (AccuPyc 1340 – Micromeritics). The specific surface area of each powder was obtained by N_2_-adsorption (ASAP 2020 – Micromeritics) and BET analysis [14] of the adsorption isotherm. The average diameter, d_mean_, of the primary particles was assessed using the following formula [15]:
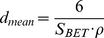
(1)


**Table 1:** Characteristics of the precursor powders.

Precursor powder name	AS21	AS11	AS12
**Al/Si molar ratio**	2∶1	1∶1	1∶2
**Specific surface area**	412.8±1.9 m^2^/g	366.2±1.2 m^2^/g	224.2±0.5 m^2^/g
**True density**	2.912±0.019 g/cm^3^	2.707±0.011 g/cm^3^	2.575±0.017 g/cm^3^
**Mean particle size**	5.0±0.1 nm	6.1±0.1 nm	10.4±0.1 nm
**ζ-potential (pH 6.8)**	−33.1±0.3 mV	−34.8±2.0 mV	−33.1±1.3 mV

The name of each precursor powder refers to its Al/Si molar ratio, as shown in the table.

where *S_BET_* is the specific surface area of the powder. This geometrical estimation of the particle size is justified as TEM studies on similar xerogels have shown that the particles are non-porous and spherical in shape [13], which is an underlying assumption of the equation. The surface charge of the particles was examined via ζ-potential measurements (Zetasizer – Malvern) in a 0.001 M KCl electrolyte with a pH adjusted to 6.8 with 0.01 M NaOH and 0.01 M HCl (same pH as in the buffer solution used in the drug release measurements described below).

### Geopolymers

**Figure 1:**
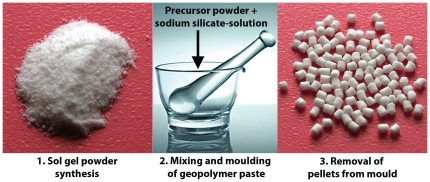
Geopolymer pellet synthesis. 1) First, precursor powders are made in a sol-gel process. 2) Thereafter, the powder is mixed with a sodium silicate solution to form a paste that is transferred to moulds and left to cure. For drug release experiments oxycodone HCL is added to the paste before moulding. 3) After curing the pellets are removed from the mould. The particular pellets in panel 3 have the dimensions Ø: 1.5 mm · h: 1.5 mm and contains oxycodone.

Fig. 1 illustrates the synthesis of geopolymer pellets. Briefly; a sodium silicate solution was prepared by adding 0.2 g NaOH per ml to a commercial sodium silicate solution containing 10.6% NaOH and 26.5% SiO_2_ (Sigma-Aldrich). Three different types of geopolymers were produced solely for material characterization by mixing the prepared sodium silicate solution with each of the sol-gel derived powders (see Table 2). The liquid to powder ratio was 1 ml/1 g in all preparations. The pastes were mixed in a mortar by hand before transferred into moulds with the dimensions Ø: 6 mm · h: 12 mm [16], and left to cure for 5 days at room temperature before taken out to dry. The sample name of each geopolymer refers to the Al/Si molar ratio of its precursor powder (e.g., GP21 was produced from a precursor powder with an Al/Si molar ratio of 2∶1).

**Table 2:** Characteristics of geopolymer samples.

Sample name of Geopolymer	GP21	GP11	GP12
**Al/Si molar ratio (and sample name) of precursor powder**	2∶1(AS21)	1∶1(AS11)	1∶2(AS12)
**Compressive strength**	20.4±4.0 MPa	39.2±5.4 MPa	10.2±2.3 MPa
**Specific surface area**	5.04 m±0.01 m^2^/g	5.09±0.01 m^2^/g	0.75±0.01 m^2^/g
**Pore volume (in pores <117 nm)**	0.0112 cm^3^/g	0.0106 cm^3^/g	0.0043 cm^3^/g
**True density**	2.142±0.002 g/cm^3^	2.207±0.012 g/cm^3^	2.223±0.002 g/cm^3^
**ζ-potential (pH 6.8)**	−49.4±0.9 mV	−54.2±1.0 mV	-61.7±0.3 mV

The name of each geopolymer sample refers to the Al/Si molar ratio of its precursor powder.

The obtained geopolymers were examined with XRD, He-pycnometry, N_2_-adsorption/desorption, and ζ-potential measurements. The mechanical strength was measured in compression mode (Autograph AGS-H universal testing machine - Shimadzu). For the latter measurements, 7 cylinders of each composition were tested. Remnants from the compression tests were used to study the densities, surface areas and porosities of the compositions. The pore size distribution and pore volume of each composition were calculated using density functional theory (DFT) [17], [18]. Left over pieces were also grinded to produce samples for XRD and ζ-potential analysis. ζ-potential measurements on the geopolymers were performed as described above.

### *In vitro* drug release

In the production of the drug carrying vehicles, geopolymer pastes were prepared as described above but with the addition of 0.02 g oxycodone HCl to each gram of precursor powder and casted to form cylindrical pellets with the dimensions Ø: 1.5 mm · h: 1.5 mm (Fig. 1, panel 3). These samples were left to cure for 5 days, either at room temperature or at 60°C, before removed from the moulds and left to dry at 60°C, see Table 3. The release measurements were performed in a USP-2 dissolution bath, 50 rpm, 37°C (AT7 Smart, Sotax) according to the *U.S. Pharmacopeia* [19]. 600 mg of pellets (each pellet weighing on average 4.4 mg) were placed in each vessel, containing 500 ml of 50 mM phosphate buffer of pH 6.8 (same pH as in the intestines where the primary drug uptake is supposed to occur). Sample aliquots of 3 ml were withdrawn with regular intervals and the concentration of oxycodone was measured with UV-vis spectroscopy at 224 nm (UV-2650pc – Shimadzu). Furthermore, sample DR21-60 was subjected to release measurements in 40vol% ethanol, and sample DR11-60 was used for release in 0.1 M HCl (pH 1.0) to simulate release in different types of relevant environments. The 40vol% ethanol release medium is often used to assess the risk of dose dumping associated with co-intake of controlled release formulations with strong spirits [20]. The acidic pH 1.0 release medium is described by the *U.S. Pharmacopeia* to be used in release measurements to simulate release from controlled release formulations in the stomach.

**Table 3:** Names and synthesis conditions of drug containing geopolymer samples.

Sample name ofdrug-containinggeopolymer	DR21-RT	DR11-RT	DR12-RT	DR21-60	DR11-60	DR12-60
**Al/Si molar ratio** **(and sample name)** **of precursor powder**	2∶1(AS21)	1∶1(AS11)	1∶2(AS12)	2∶1(AS21)	1∶1(AS11)	1∶2(AS12)
**Curing temperature**	Room temp.	Room temp.	Room temp.	60°C	60°C	60°C

To investigate the influence of pellet size on the drug release rate, an additional set of DR11-RT-pellets were produced with the dimensions Ø: 0.5 mm · h: 1.0 mm. The drug release from these smaller pellets was measured in the 50 mM phosphate buffer at pH 6.8.

## Results and Discussion

### Precursor powders

**Figure 2:**
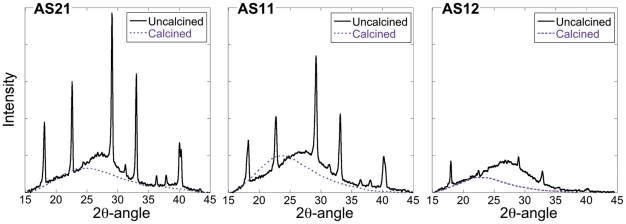
XRD patterns for the sol-gel derived precursor powders before and after calcination at 800°C.

The obtained precursor powders were completely white (Fig. 1, panel 1) and the corresponding XRD patterns for each powder displayed peaks associated with crystalline SiO_2_ prior to the calcination, although the peak intensities decreased with increasing Si content, see Fig. 2. It is also evident from Fig. 2, that the three powders adopted a clear amorphous structure after the calcination, which is desired as it increases the reactivity of the powders [21]. The measured specific surface areas and densities of the powders increased with aluminum content while the calculated mean sizes of the primary particles decreased, see Table 1. The primary particles in AS11 and AS21 were almost equal in size (∼5–6 nm) while the AS12 particles were considerably larger (∼10 nm). These findings are in agreement with earlier TEM studies [13]. No significant difference in surface charge was seen between the powders that all had a ζ-potential of about -33 mV, see Table 1.

### Geopolymers

When the precursor powders were mixed with the sodium silicate solution to form geoplymers, AS11 and AS21 readily dissolved to form cohesive pastes that were easy to transfer to the moulds. AS12 did not react as easily and the obtained paste behaved more like toothpaste containing dispensed grainy particles. Upon hardening, composition GP12 shrank and the casted cylinders were easy to retrieve from the moulds, while composition GP21 increased in volume. No significant volume change was observed for composition GP11 after hardening. The three different geopolymers were all clear white and appeared slightly translucent which is consistent with an inherent nanostructure and mesoporosity (mesopores: pores with a diameter between 2 nm and 50 nm) [22], [23].

**Figure 3:**
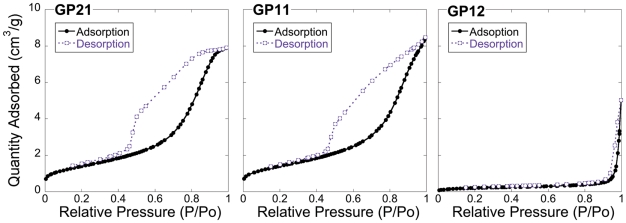
N_2_ isotherms for the geopolymer samples.

**Figure 4:**
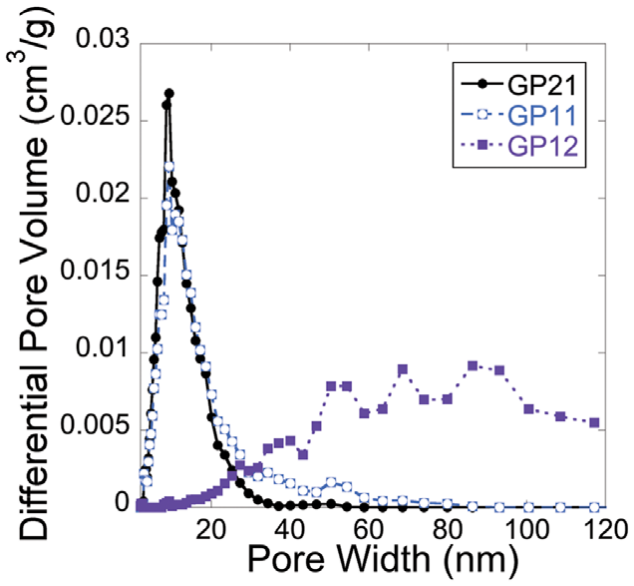
Pore size distributions in the geopolymer samples.

The N_2_-isotherms for the geopolymer compositions are presented in Fig. 3. It was evident from the adsorption measurements that GP12 had a relatively compact structure with lower pore volume and smaller specific surface area compared to the other compositions, as shown in Table 2. The shape of the wide hysteresis for GP21 and GP11 is consistent with irregular interconnected and slit like mesopores [24] and the steep drop in the desorption branch at a partial nitrogen pressure p/p_0_ ≈0.46 suggests a presence of ink-bottle shaped pores with necks smaller than ∼5 nm [25]. The saturation plateau reached at p/p_0_ ≈0.9 for GP21 represents a complete filling of condensed gas in the structure and is indicative of a lack of larger pores above the mesoporous range. The pore size distributions of the three geopolymers are seen in Fig. 4. Here a distinct difference can be seen between the compositions; the majority of pores in GP21 and GP11 are gathered in a narrow size region around 10 nm while the pores in GP12 have a wide size distribution mainly in the microporous range (above 50 nm). It is clear from Fig. 4 that GP21 had the most distinct pore size distribution with all pores in a range between 2 nm and 35 nm while GP11 contains pores with sizes up to 85 nm. In addition to having the highest accessible pore volume, GP21 also seemed to have the highest inaccessible pore volume, judging from the fact that it had the lowest true density as shown in Table 2 (true density is the skeletal density of the material *including* inaccessible pores).

AS11 reacted rapidly with the sodium silicate solution to form geopolymers within an hour after mixing while the other powders needed substantially longer times to set; AS21 had the slowest setting rate and needed several days to set. The compressive strength of the compositions differed significantly; GP11 had the highest compressive strength, twice as high as for GP21 and four times that of GP12, see Table 2. The compressive strength of GP11 can be compared with the strength of high strength concrete but is several times lower that that of teeth. The relatively low compressive strength of GP12 may be related to a limited polymerization as AS12 did not dissolve as easily as the other powders in the sodium silicate solution. The higher strength of GP11 compared to GP21 may be explained by the higher porosity in GP21, and also by the higher Si content in GP11 which clearly increased the reactivity of this powder and shortened the setting time. The high reactivity of GP11 may have caused a more complete and homogeneous geopolymerization, as compared to the other samples.

**Figure 5:**
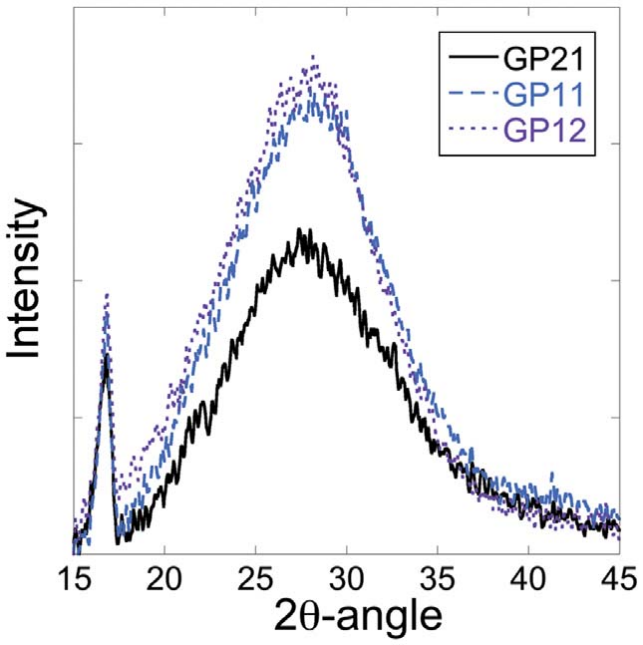
XRD pattern for the geopolymer samples. The broad hump in the pattern is characteristic for amorphous geopolymer while the peak left to the bump is indicative for a formation of a crystalline phase.

The XRD patterns for all three geopolymers contained the characteristic hump corresponding to the amorphous structure in the geopolymer [10], see Fig. 5. There was also a distinct peak in the patterns indicating the presence of a crystalline phase, but it is hard to make a correct determination of this phase from a single XRD peak. Yet, this peak was not seen in the XRD patterns for any of the precursor powders, indicating that the formation of this phase was induced by the reaction between the sodium silicate solution and the precursor powders. Unlike the situation for the precursor powders, the ζ-potential measurements of the geopolymers revealed a difference between the compositions where the negative surface charge increased with Si content, see Table 2. The surface charge was significantly larger for all geopolymers compared to their corresponding precursor powder with GP12 possessing the most negative ζ-potential at -61.7±0.3 mV. The difference between the geopolymer samples may be attributed to the difference in numbers of silanol (SiOH) and aluminol (AlOH) groups present on the surfaces [26] where the amphoteric alumina generally is protonated and carries a positive charge at pH 6.8 while the silica species are negatively charged [27]. The profound electronegativity of the geopolymer walls enables electrostatic interaction with ionized molecules integrated in the porous matrixes. As oxycodone is a weak base with pK_a_ of 8.5 [28], most of the species in solution will be protonated and carry a positive charge at pH 6.8 according to the Henderson-Hasselbalch equation [29]. This opposite charge promotes an attraction between oxycodone and the geopolymer wall and is thus expected to affect the release profiles.

### *In vitro* drug release

**Figure 6:**
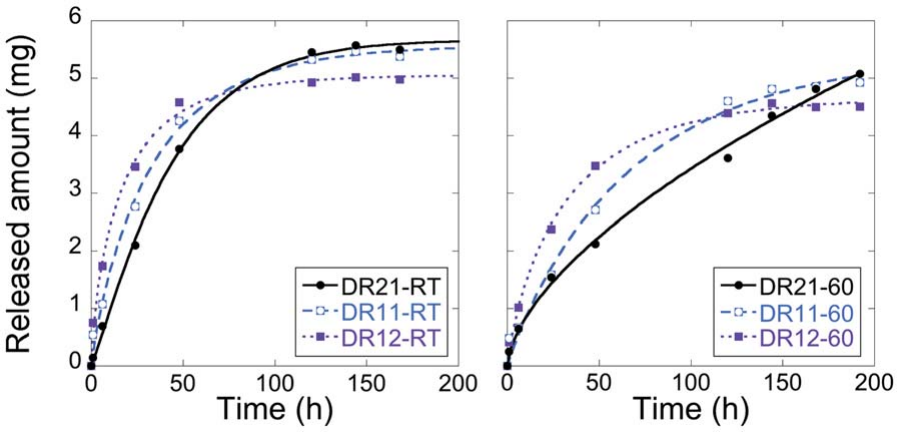
Release profiles in pH 6.8 for the oxycodone carrying geopolymer samples. Each curve show the total release of oxycodone from roughly 135 pellets (weighing in total 600 mg) with the dimensions Ø: 1.5 mm · h: 1.5 mm.

**Figure 7:**
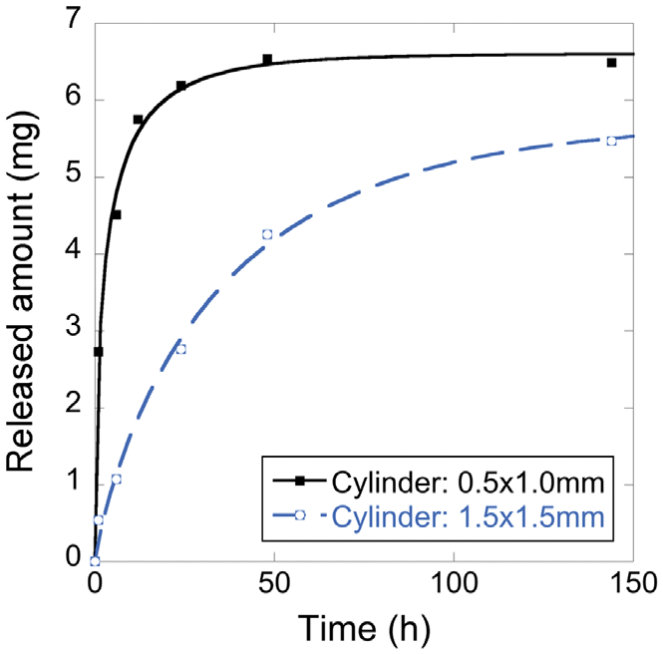
Influence of pellet size on drug release profile. Oxycodone is released in pH 6.8, from DR11-RT pellets of two different dimensions: (i) Ø: 0.5 mm · h: 1.0 mm and (ii) Ø: 1.5 mm · h: 1.5 mm

Continuous and sustained releases of oxycodone for up to 6 days were obtained for all drug-carrying geopolymer compositions with the dimensions Ø: 1.5 mm · h: 1.5 mm, see Fig. 6. This is substantially slower compared to a previous study where fentanyl was released from bodies of similar size but comprised of metakaolin-based geopolymers [9]. In the mentioned study, most of the fentanyl was released after 24 h, and this difference in release rate may be attributed to the more limited porosity together with a more distinct mesoporous structure of the geopolymers in the present study. A sustained release for several days is obviously not desirable in this context as the pellets would have left the intestines long before the release is complete. But the profound retardation of the drug release from the geopolymer structures opens up for precise control of the sustained release rates, simply by adjusting the size of the pellets and thereby affecting the diffusion length in the structures. This is clear from Fig. 7, where drug release from pellets of two different sizes is compared. The figure shows that decreasing the pellet size increases the release rate, and a clinically relevant release period of about 12 h is obtained with pellets of the size Ø: 0.5 mm · h: 1.0 mm (88% of the total release was achieved after 12 h). Fig. 7 also shows that the fraction of the drug that is trapped in closed pores (i.e. that can not be released) is smaller for the smaller pellets, which is expected due to the larger surface area of the smaller pellets.

As shown in Fig. 6, it was observed that the oxycodone release rate decreased with increasing Al/Si ratio, observed for both investigated setting temperatures. This is consistent with the fact that the pore sizes also decreased with increasing Al/Si ratio (see Fig. 4); it is logical that drug diffusion through the geopolymer, and therefore also drug release, is slower in a system of smaller pores [30]. Increasing aluminum content resulted in a more linear release profile, and this linear release should also be attributed to the shift towards smaller pores in the aluminum rich compositions, since it has been shown that polymeric systems with pore diameters in a range comparable with the size of the drug molecules enables zero order release [31]. The present work shows that linear release profiles can be achieved with the presented geopolymer system, simply by tuning the Al/Si ratio and curing temperature. This is a large benefit compared to previously described dosage forms of clay-derived geopolymers where the release profiles were more similar to the ones of the DR12 samples.

It was clear from the release measurements that a setting temperature of 60°C resulted in slower release, as compared to setting at room temperature; this was observed for all three investigated Al/Si ratios of the precursor powder. This may be a result of a capillary contraction of the structures as the water leaves the geopolymers more easily at this temperature, forcing formed pores to shrink before complete cementation of the structures. After the release of oxycodone had leveled out, the buffer concentration was increased fourfold to examine if more oxycodone could be released by screening the charge of the geopolymer walls (examined after 200 h of release, for the samples that had cured at room temperature). The increase in buffer concentration did not result in any additional release of oxycodone, showing that a buffer concentration of 50 mM was sufficient to release all oxycodone (except the fraction confined in the closed pores). After 200 h of release, 5.6 mg Oxycdone had been released from DR21-RT pellets of the larger size, which corresponds to roughly 80% of the total amount of incorporated oxycodone (exact calculations of released fractions were precluded by the difficulty in assessing the amount of water entrapped in the structure after synthesis).

**Figure 8:**
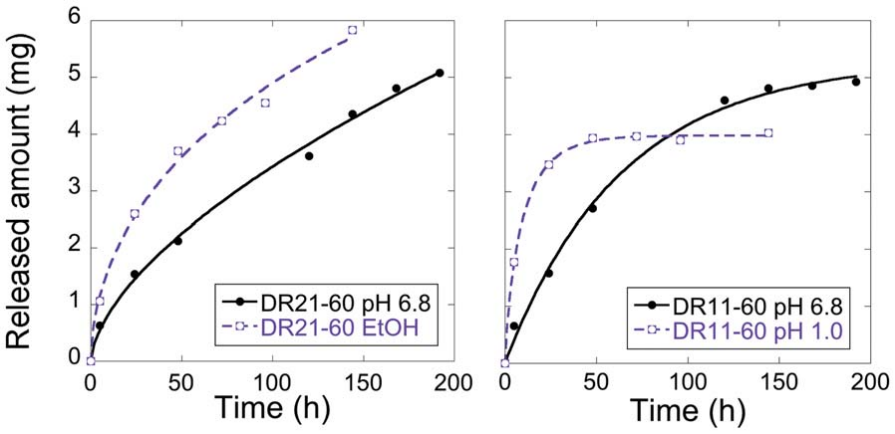
Release profiles in pH 6.8 and in 40 vol% ethanol. Comparison of release profiles for DR21-60 in pH 6.8 and in 40% ethanol, and for DR11-60 in pH 6.8 and pH 1.0 (pellet dimensions Ø: 1.5 mm · h: 1.5 mm).

DR21-60 was also subjected to release measurements in 40vol% ethanol to examine if alcohol affects the release rate. As seen in Fig. 8, where the release of oxycodone in ethanol is compared with the release in phosphate buffer, alcohol increases the release rate. The increase at this extreme condition seems however relatively limited, i.e. less than twofold. 40% ethanol is an extremely high concentration that only is possible to achieve under very limited time if the pellets is to be swallowed together with strong sprits. The alcohol will be diluted by the gastric juice in the stomach and the concentration will rapidly decline once the liquid enters the small intestine where the uptake of alcohol is rapid [20]. Under more realistic conditions with lower alcohol concentrations, the effect of alcohol on the release should be negligible as seen for other systems [20], [29].

The release of oxycodone from DR11-60 was also examined at pH 1.0, to see how this acidic condition affected the release. As shown in Fig. 8, the release rate is substantially higher at pH 1.0 compared to the release at pH 6.8, in agreement with earlier findings [9]. Interestingly, the release at pH 1.0 suddenly ceases after about 48 h even though only 4 mg has been released, compared to the total release of 5 mg after 144 h at pH 6.8. This may be explained by a collapse of the porous structure due to rearrangement of the geopolymer; it has been shown previously that geopolymers can dissolve and condensate into new forms under strong acidic conditions [11]. Fig. 8 suggests that the presently investigated geopolymers break down and recondensate at pH 1.0, causing some of the oxycodone to become trapped in closed pores. These negative effects of low pH on the release can, however, be overcome by administration of the pellets in enteric-coated formulations.

Using geopolymers as drug delivery vehicles introduces a new concept in the pharmaceutical sciences, and such vehicles have so far not been tested *in vivo.* Thus, it remains to be investigated if any negative side effects are linked to the proposed carrier material. The small size of the pellets used here and the lack of sharp edges on these, similar to other non-degrading carriers [29], [32], make it unlikely the pellets would mechanically affect the epithelium of the intestines due to sharp entities on the carrier surface. If necessary, the pellets could be molded into spherical shape. Furthermore, geopolymers have previously been suggested as material in implant applications and been shown to both have bioactive properties and low leakage of ions [33], suggesting that the material is non-toxic.

In order to further establish that the proposed concept can be condensed into actual use, *in vivo* studies of a final product should be performed. Such studies should include pellets compacted with conventional tablet excipients and administered as enteric-coated tablets, or pellets that are administered in enteric-coated capsules, to ensure tablet disintegration and drug uptake in the small intestine.

### Conclusions

In the present study, fully synthetic geopolymers were obtained by the reaction of a sodium silicate solution with sol-gel synthesized aluminosilicate nanoparticles. Precursor nanoparticles with three different Al/Si molar ratios were investigated: 2∶1, 1∶1 and 1∶2. By altering the Al/Si molar ratio of the nanoparticles, several properties of the corresponding geopolymers could be adjusted: for example, mechanical strength, setting time and swelling/shrinking behavior during setting. A particularly interesting result is the fact that the pore size distribution was narrowed as the Al/Si molar ratio was increased. For the highest investigated Al/Si molar ratio, 2∶1, the entire pore volume was within the mesoporous range (all pores were between 2 nm and 35 nm). The geopolymers were used as drug carrying vehicles for sustained release of the opioid oxycodone. A profound retardation of the release was observed *in vitro,* and it was possible to obtain an almost linear release profile from the aluminum rich compositions, which is a very desirable property for sustained release formulations. By tuning the size of the pellets, it was possible to obtain a release period of about 12 h, which is a clinically relevant release time. The mechanical strength makes the obtained geopolymers difficult to crush upon accidental chewing, and the geopolymers are therefore a safe and attractive material for controlled release of drugs with narrow therapeutic window, such as highly potent opioids. This study has focused on the release of opioids for treatment of chronic pain, but the presented geopolymers can also be used for drug administration in other clinical indications where a constant or sustained delivery of drugs is desirable.
